# Increased Risk of Acute Angle Closure in Retinitis Pigmentosa: A Population-Based Case-Control Study

**DOI:** 10.1371/journal.pone.0107660

**Published:** 2014-09-15

**Authors:** Yu-Chieh Ko, Chia-Jen Liu, De-Kuang Hwang, Tzeng-Ji Chen, Catherine J. Liu

**Affiliations:** 1 Department of Ophthalmology, Taipei Veterans General Hospital, Taipei, Taiwan; 2 Faculty of Medicine, National Yang-Ming University School of Medicine, Taipei, Taiwan; 3 Institute of Clinical Medicine, School of Medicine, National Yang-Ming University, Taipei, Taiwan; 4 Division of Hematology and Oncology, Taipei Veterans General Hospital, Taipei, Taiwan; 5 Institute of Public Health, School of Medicine, National Yang-Ming University, Taipei, Taiwan; 6 Department of Ophthalmology, Taichung Veterans General Hospital, Taichung, Taiwan; 7 Department of Family Medicine, Taipei Veterans General Hospital, Taipei, Taiwan; University of Florida, United States of America

## Abstract

**Purpose:**

To investigate the association between retinitis pigmentosa (RP) and acute angle closure during a 15-year follow-up period.

**Methods:**

Using the Taiwan Longitudinal Health Insurance Database 2000, we identified 382 RP patients based on the diagnostic code of RP (International Classification of Diseases, 9^th^ Revision, Clinical Modification (ICD-9-CM) 362.74) made during 1996–2010, excluding subjects under age of 20 years at diagnosis or subjects undergoing lens extraction before the index date. The control group included 3820 randomly selected non-RP subjects matched with the RP patients in age, gender and the index date of diagnosis. The incidence of acute angle closure during the study period was observed based on an ICD-9-CM code of 365.22. Cochran-Mantel-Haenszel test was used to determine the odds ratio (OR) of having acute angle closure in RP patients.

**Results:**

The mean age at the diagnosis of RP was 51.1years (standard deviation [SD] 16.7). Acute angle closure occurred in 5 RP patients (1.3%) and in 15 controls (0.4%). The mean age with the acute angle closure was 53.3 years (SD 8.0) in RP patients and 64.6 years (SD 8.4) in controls (*P* = 0.015). After adjusting for age, gender and comorbid disorders, RP patients had 3.64-fold (95% confidence interval [CI], 1.29–10.25, *P*<0.001) greater odds of having acute angle closure. After stratification for gender and age, the risk of acute angle closure in RP was higher in patients under age of 60 years (adjusted OR 11.84; 95% CI, 2.84–49.48) and male patients (adjusted OR 19.36; 95% CI, 3.43–109.40)(both *P* = 0.001).

**Conclusions:**

RP patients had increased risk of acute angle closure than controls. Contrary to the fact that angle closure disease is more prevalent in elderly females in general population, acute angle closure attack occurred earlier in life and the risk was higher in males among RP patients.

## Introduction

The association between retinitis pigmentosa (RP) and glaucoma was first proposed in 1862, followed by dozens of case reports [1], [2]. The diagnosis of glaucoma in subjects with RP may be confounded by coexistent RP-related changes such as retinal nerve fiber layer defect and visual field defect [3], [4]. An elevated intraocular pressure (IOP) confirmed the diagnosis of glaucoma in most of the reported cases, but the status of the drainage angle was mostly not evaluated. Angle closure glaucoma should be suspected in many of the above mentioned cases because an acute highly elevated IOP or absolute glaucoma were common presenting features [1]. A hospital-based study in China found primary angle closure glaucoma (93.8%) as the predominant form among the 2.3% of RP patients with coexistent glaucoma (32 glaucoma patients out of 1400 RP cases) [5].

RP is a major cause of blindness in adults [6]. IOP elevation may aggravate the visual impairment in RP patients with preexisting retinal vasculopathy and optic nerve dysfunction [4], [7], [8]. Acute angle closure, the major risk factor for blindness among angle closure eyes, may cause particularly devastating damage to RP patients [9]. Angle closure related IOP elevation is preventable by timely intervention to widen the drainage angle in susceptible eyes [10]. Therefore, understanding the association of angle closure disease and RP may help preserve visual function in these patients. However, the prevalence of angle closure disease in RP patients was almost unknown.

This study aimed to explore the association between RP and acute angle closure in a population-based cohort. A nationwide health insurance database with high representativeness of population in Taiwan was used to retrieve cases of RP to observe the event of acute angle closure. Considering the high prevalence of angle closure disease in Chinese [11], only subjects with acute angle closure were included in this study to avoid overestimating the association between RP and angle closure disease by misclassifying RP-related optic neuropathy concurrent with age-related angle narrowing as angle closure glaucoma. The subjects identified were therefore those suffering from angle closure process requiring timing intervention to prevent rapid vision deterioration.

## Materials and Methods

### Database

This was a nationwide population-based retrospective case-control study. Taiwan began the National Health Insurance program in 1995 which covers >98% of the current 23 million residents. The National Health Insurance Research Database (NHIRD) consisted of the registration files and claim data of all the enrollees. We used the Longitudinal Health Insurance Database (LHID2000), a data subsets constructed from NHIRD for research purpose. LHID2000 was created by systematically and randomly selecting one million subjects, who were covered by the insurance program during 2000 and contains all their de-identified enrolment files, claim data and the registry for prescription drugs from 1996 to 2010. There were no differences in age, gender, or healthcare costs between the sampled group and all the enrollees [12]. The accuracy of the claims data of the NHIRD was properly monitored and validated [13], [14], and hundreds of studies based on this dataset have been published in peer-reviewed journals, including ophthalmology-related studies and those focused on angle closure glaucoma or retinitis pigmentosa [15]–[17].

Because the dataset consisted of de-identified data released for research, the study was exempt from full review according to the institutional review board of our institute.

### Study Subjects

We identified 382 subjects from the LHID2000 with the diagnosis code of RP (International Classification of Diseases, 9^th^ Revision, Clinical Modification (ICD-9-CM) 362.74) made between 1 January 1996 and 31 December 2010, excluding subjects under the age of 20 years at diagnosis (n = 30) and subjects having undergone lens extraction with a diagnostic code of 366.5 or V43.1 before the index date (n = 0). The index date for a RP patient was the date when the disease was first coded.

For the potential systemic involvement in RP, we evaluated the comorbid diseases, including diabetes mellitus, hypertension, coronary artery disease, dyslipidemia, cerebrovascular disease, asthma, autoimmune disease, malignancies, liver cirrhosis, and chronic kidney disease during the follow-up period as a potential confounding situation. Selected medical comorbidities were counted only if the condition occurred either in an inpatient setting or in more than 2 ambulatory care claims during the study period.

### Control cohort

The control cohort was selected randomly from the same database in a ratio of 10 controls to each RP patient who were matched in age, gender and index date as the subjects with RP. Subjects had undergone lens extraction with a diagnostic code of 366.5 or V43.1 before the index date were excluded.

### Angle closure glaucoma event measurement

Episodes of acute angle closure were identified by having an ICD-9-CM code of 365.22 (acute angle closure glaucoma). This episode could occur before or after the diagnosis of RP.

### Statistical analysis

Extraction and computation of data were performed using the Perl programming language. Microsoft SQL Server 2008 (Microsoft Corp., Redmond, WA, USA) was used for data management and computing. Statistical analysis was performed using SPSS statistical software (version 15.0.0, SPSS, Inc. Chicago, IL, USA). All data were expressed as the frequency (percentage) for categorical data or as mean and standard deviation (SD) for continuous data. We compared the distribution of demographic factors and medical comorbid disorders between the RP and control groups using the chi-square test or Fisher's exact test for categorical variables and Mann-Whitney U test for continuous variables. The odds ratio (OR) of having angle closure attack in subjects with RP was evaluated by Cochran-Mantel-Haenszel test with stratification for age and gender, and adjustment for comorbiditis, age and gender.

## Results

We assessed data of 382 subjects with RP and 3820 age and gender matched controls. The mean age at the diagnosis of RP was 51.1years (SD 16.7) and 45.3% of the subjects were male. Subjects with RP had a higher prevalence of the following comorbid diseases than the control cohort: diabetes mellitus, hypertension, coronary artery disease, and chronic renal disease (Table 1). In total, 20 subjects suffered from acute angle closure, including 5 RP patients (1.3%), and 15 subjects in the control cohort (0.4%). Most subjects had the acute angle closure after the index date, except for 1 RP subjects and 5 control subjects who had acute angle closure 1.2 years and 5.0 years (range 0.7–11.8) before, respectively. Acute angle closure occurred at a younger age in RP patients than controls (53.3(SD 8.0) and 64.6(SD 8.4), respectively, *P* = 0.015). Table 2 revealed the crude and adjusted odds ratio of having acute angle closure in subjects with RP with the adjustment for age, gender and comorbid diseases. Compared with the control cohort, subjects with RP had a 3.64-fold (95% confidence interval [CI], 1.29–10.25, *P*<0.001) greater odds of having acute angle closure. After stratification by age and gender, increased risk of acute angle closure in RP patients was observed in subjects under age of 60 years (adjusted OR, 11.84; 95% CI, 2.84–49.48, *P* = 0.001) and male subjects (adjusted OR, 19.36; 95% CI, 3.43–109.40, *P* = 0.001).

**Table 1:** Baseline characteristics and comorbid medical disorders of patients with retinitis pigmentosa (RP) and the control cohort.

Demographic data	RP cohort *n* = 382		Control cohort *n* = 3820		*P* value*
	*n*	%	*n*	%	
**Age**					
<60 years	249	65.2	2490	65.2	1.000
≥60 years	133	34.8	1330	34.8	
**Gender**					
Male	173	45.3	1730	45.3	1.000
Female	209	54.7	2090	54.7	
**Comorbid diseases**					
Diabetes mellitus	84	22.0	619	16.2	0.004
Hypertension	135	35.3	1093	28.6	0.006
Coronary artery disease	10	2.6	48	1.3	0.030
Autoimmune disease	29	7.6	231	6.0	0.232
Malignancies	7	1.8	105	2.7	0.289
Liver Cirrhosis	10	2.6	66	1.7	0.213
Chronic kidney disease	49	12.8	302	7.9	0.001
Cerebrovascular disease	53	13.9	415	10.9	0.075
Dyslipidemia	90	23.6	821	21.5	0.350
Asthma	55	14.4	429	11.2	0.065

* Analyzed by Chi-square test or Fisher's exact test.

**Table 2:** Incidence and risk of acute angle closure in patients with retinitis pigmentosa (RP) and the control cohort.

Subjects with acute attack	RP cohort (*n* = 382)		Matched cohort (*n* = 3820)		Odds ratio (95% CI)*	*P* value	Adjusted odds ratio (95% CI)* †	*P* value
	n	%	No.	%				
**Total**	5	1.31	15	0.39	3.36 (1.22–9.31)	0.019	3.63 (1.29–10.24)	0.015
**Age**								
<60 years	4	1.61	5	0.20	10.15 (2.52–40.82)	0.001	11.97 (2.85–50.30)	0.001
≥60 years	1	0.75	10	0.75	0.91 (0.12–7.09)	0.927	0.92 (0.11–7.46)	0.940
**Gender**								
Male	4	2.31	4	0.23	13.63 (3.02–61.39)	0.001	19.59 (3.45–111.13)	0.001
Female	1	0.48	11	0.53	0.83 (0.11–6.44)	0.861	0.75 (0.09–5.95)	0.783

CI = confidence interval.

* Analyzed by Cochran-Mantel-Haenszel test.

† Adjusted for comorbid diseases listed in table 1 and all other factors in this table.

## Discussion

This is the first population-based study to determine the association of acute angle closure and RP. The prevalence of RP in this cohort (0.0382%) is similar to the reported worldwide prevalence of 1∶4000 [6], [18], [19]. Comparing with the respective controls, acute angle closure occurred more frequently in RP at a younger age and the risk was higher in male patients. The highest risks of acute attack in RP are adults in the working age group.

Patients with RP continuously face a slowly progressive loss of vision, acute angle closure may aggravate the visual loss over a short period of time in these patients. However, the impact of acute angle closure on RP patients was not clearly understood. Our study identified 5 acute angle closure episodes (1.3%) in 382 RP patients during a 15-year period, much more than the rate noted in the control subjects (0.4%). Our finding was in line with a hospital-based study in Canada. Comparing with the prevalence of primary angle closure glaucoma (0.07%) in a community based population, the authors found a higher prevalence of it in patients with RP; 5 cases (1.03%) with coexistent angle closure glaucoma in 388 RP patients aged over 40 years during a 13-year period [20]. Of the 3 cases with detailed history, two had episodes of acute attack. Another hospital-based case series in China found angle closure glaucoma was the predominant form (93.8%) in RP patients with coexistent glaucoma [5]. All these findings support a strong association between RP and angle closure glaucoma.

RP is a hereditary retinal degenerative disease affecting primarily the photoreceptor rods. Syndromic RP refers to those associated with additional ocular or non-ocular findings. The hereditary nature of RP and current understanding about these two diseases do not suggest angle closure disease as a risk factor of RP. Contrarily, the associated ocular findings in RP, such as nanophthalmos, zonular insufficiency, or ectopic lentis, may explain the increased prevalence of angle closure glaucoma in RP [21]–[27].

RP has been reported to be associated with nanophthalmos as a rare syndrome [21]–[24]. The biometric characteristics of nanophthalmos predispose these eyes to developing angle closure glaucoma at a younger age [28]. Genetic mutation responsible for this syndrome has been identified but the exact pathologic mechanism responsible for defective ocular development and retinal dysfunction is not clear [23], [24].

Coexistent nanophthalmos in RP could not explain most of the angle closure diseases in RP. In Badeeb *et al*'s cohort, RP patients suffering from angle closure glaucoma did not have high hyperopia. They had a mean axial length of 23.4 mm (SD 0.49) as normal population, but a thicker and more anteriorly positioned lens as those with primary angle closure glaucoma [20]. Zonular instability is prevalent in RP patients, which might result in anterior displacement of the lens and angle narrowing [25], [29]. Moreover, ectopia lentis and lens subluxation have been reported as causes of angle closure glaucoma in RP patients after excluding systemic disorders that might be associated with ectopia lentis [26], [27]. Therefore, angle closure glaucoma may be caused by thickened, anteriorly displaced, even luxated lens in RP subjects with a normal axial length.

Primary angle closure glaucoma most frequently affects elderly Chinese females because of their constitutional biometric characteristics and aging-related changes in the anterior chamber angle [30], [31]. Our finding that acute angle closure occurred at a younger age and more frequently in male in RP suggests a pathological process distinguished from that in primary angle closure glaucoma. Zonular insufficiency, which was observed in 18.8% of RP patients undergoing lens extraction at an average age of 49 years, may account for the early acute angle closure in RP [25]. Timely lens extraction may prevent the acute attack in these patients but careful preoperative assessment of lens stability is important for preparing adjunctive method to maximize postoperative lens stabilization [25], [32]. No gender difference was observed in subjects with RP suffering angle closure glaucoma or zonular insufficiency [21]–[27]. Further study with more cases is needed to verify the male predisposition of acute angle closure in RP.

In this study, RP subjects had a higher prevalence of diabetes, hypertension, coronary artery disease, and chronic renal disease than controls. This could be related to non-ocular disorders found in about 20–30% of RP patients [6]. The renal disorders and obesity associated with RP can lead to hypertension and coronary artery disease. However, diabetes was not frequently reported in syndromic RP [33]. To avoid the possible confounding effect due to mismatch in comorbid diseases between RP and controls, we adjusted all the comorbidities while estimating the risk of developing acute angle closure in RP.

The findings of this study need to be interpreted in the context of the following limitations. First, the diagnosis of RP and the comorbid diseases were dependent on the ICD-9-CM codes, which were less accurate than those obtained through standardized examinations. It is possible to misclassify cases with retinal pigmentary changes secondary to infectious, inflammatory, toxic etiology, or cone dystrophies as RP. Second, to avoid overestimating the association between angle closure glaucoma and RP caused by misclassifying RP-related optic neuropathy with concurrent age-related angle narrowing as angle closure glaucoma, only eyes with acute angle closure, which is less likely to be miscoded were selected. Whether subjects with RP are also prone to having asymptomatic angle closure glaucoma needs further evaluation. Lastly, we excluded RP patients under age of 20 years based on the regulation of Institutional Review Board. However, acute angle closure has never been reported in RP patients under this age.

In this study, acute angle closure occurred more frequently, at a younger age, and more common in male in RP patients than that in controls. The angle closure may be caused by zonular insufficiency or nanophthalmos associated with RP. It is important to integrate anterior chamber depth estimation, gonioscopy, and lens status evaluation in the management of subjects with RP. Timely intervention to widen the drainage angle may protect RP patients from aggravated damage caused by an elevated IOP.
